# Structural Similarities between Some Common Fluorophores Used in Biology, Marketed Drugs, Endogenous Metabolites, and Natural Products

**DOI:** 10.3390/md18110582

**Published:** 2020-11-23

**Authors:** Steve O’Hagan, Douglas B. Kell

**Affiliations:** 1 Department of Chemistry, The University of Manchester, Manchester M13 9PT, UK; sohagan@manchester.ac.uk; 2 Manchester Institute of Biotechnology, The University of Manchester, 131 Princess St, Manchester M1 7DN, UK; 3 Department of Biochemistry and Systems Biology, Institute of Molecular, Integrative and Systems Biology, Biosciences Building, University of Liverpool, Crown Street, Liverpool L69 7ZB, UK; 4 Novo Nordisk Foundation Centre for Biosustainability, Technical University of Denmark, Building 220, Kemitorvet, 2800 Kongens Lyngby, Denmark

**Keywords:** drugs, natural products, fluorophores, fingerprinting, similarity, cheminformatics, rdkit, Tanimoto distance

## Abstract

It is known that at least some fluorophores can act as ‘surrogate’ substrates for solute carriers (SLCs) involved in pharmaceutical drug uptake, and this promiscuity is taken to reflect at least a certain structural similarity. As part of a comprehensive study seeking the ‘natural’ substrates of ‘orphan’ transporters that also serve to take up pharmaceutical drugs into cells, we have noted that many drugs bear structural similarities to natural products. A cursory inspection of common fluorophores indicates that they too are surprisingly ‘drug-like’, and they also enter at least some cells. Some are also known to be substrates of efflux transporters. Consequently, we sought to assess the structural similarity of common fluorophores to marketed drugs, endogenous mammalian metabolites, and natural products. We used a set of some 150 fluorophores along with standard fingerprinting methods and the Tanimoto similarity metric. Results: The great majority of fluorophores tested exhibited significant similarity (Tanimoto similarity > 0.75) to at least one drug, as judged via descriptor properties (especially their aromaticity, for identifiable reasons that we explain), by molecular fingerprints, by visual inspection, and via the “quantitative estimate of drug likeness” technique. It is concluded that this set of fluorophores does overlap with a significant part of both the drug space and natural products space. Consequently, fluorophores do indeed offer a much wider opportunity than had possibly been realised to be used as surrogate uptake molecules in the competitive or trans-stimulation assay of membrane transporter activities.

## 1. Introduction

Fluorescence methods have been used in biological research for decades, and their utility remains unabated (e.g., [1,2,3,4,5,6,7,8,9,10,11,12,13,14,15,16,17,18,19,20,21,22]). Our specific interest here is in the transporter-mediated means by which small fluorescent molecules enter living cells, and our interest has been stimulated by the recognition that a given probe may be a substrate for a large variety of both influx and efflux transporters [23]. Efflux transporters are often fairly promiscuous, since their job is largely to rid cells of unwanted molecules that may have entered, although they can and do have other, important physiological roles (e.g., [24,25,26,27,28,29,30,31,32,33,34]), and are capable of effluxing a variety of fluorescent probes (e.g., [35,36,37,38,39,40,41,42]). However, given that most of these probes are contemporary, synthetic molecules, the uptake transporters for which they are substrates are nonetheless often ancient [43,44], and must have evolved in nature for other purposes. These purposes may reasonably be expected to include the uptake of endogenous metabolites in multicellular organisms [45,46,47,48,49,50,51,52,53], as well as exogenous natural products whose uptake can enhance biological fitness (e.g., [54,55]). This explanation does seems to hold well for synthetic, marketed pharmaceutical drugs [55].

Consequently, it seemed reasonable that existing fluorescent molecules, that are not specifically designed for the purpose but are taken up by biological cells, might also bear structural similarities to endogenous substrates (metabolites) and to natural products (including those from the marine environment), and potentially also to marketed drugs. Of course, some marketed pharmaceutical drugs that are transported into cells are, in fact, naturally fluorescent, including molecules such as anthracyclines [56,57,58], mepacrine (atebrin, quinacrine) [59], obatoclax [60,61], tetracycline derivatives [57,62] and topotecan [63], The same is true of certain vitamins such as riboflavin [64,65] (that necessarily have transporters, as cells cannot synthesise them), as well as certain bioactive natural products (e.g., [66,67,68]). As per this Special Issue, and in an era of increasing antimicrobial resistance [69,70,71,72], this is very much the case for novel antimicrobials, which classically come from natural products (e.g., [73,74,75,76,77,78]). If so, they might then serve as surrogate transporter substrates for them. Indeed, there are examples—so-called fluorescent false neurotransmitters—where such fluorescent analogues of natural substrates have been designed precisely for this purpose (e.g., [79,80]). The aim of the present work was to assess the extent to which this kind of structural similarity between (i) common fluorophores used in biology and (ii) other molecular classes (endogenous mammalian metabolites, marketed pharmaceutical drugs, and known natural products) might be true. It is concluded that in structural terms, common fluorophores do indeed overlap drug space significantly, and we offer an explanation based on the consonance between aromaticity, conjugated π-bonds, and fluorescence. Such fluorophores might then lend themselves readily to high-throughput assays in which their uptake and/or efflux are studied in the presence and absence of ‘competing’ drugs. A preprint is available [81].

## 2. Results

**Figure 1:**
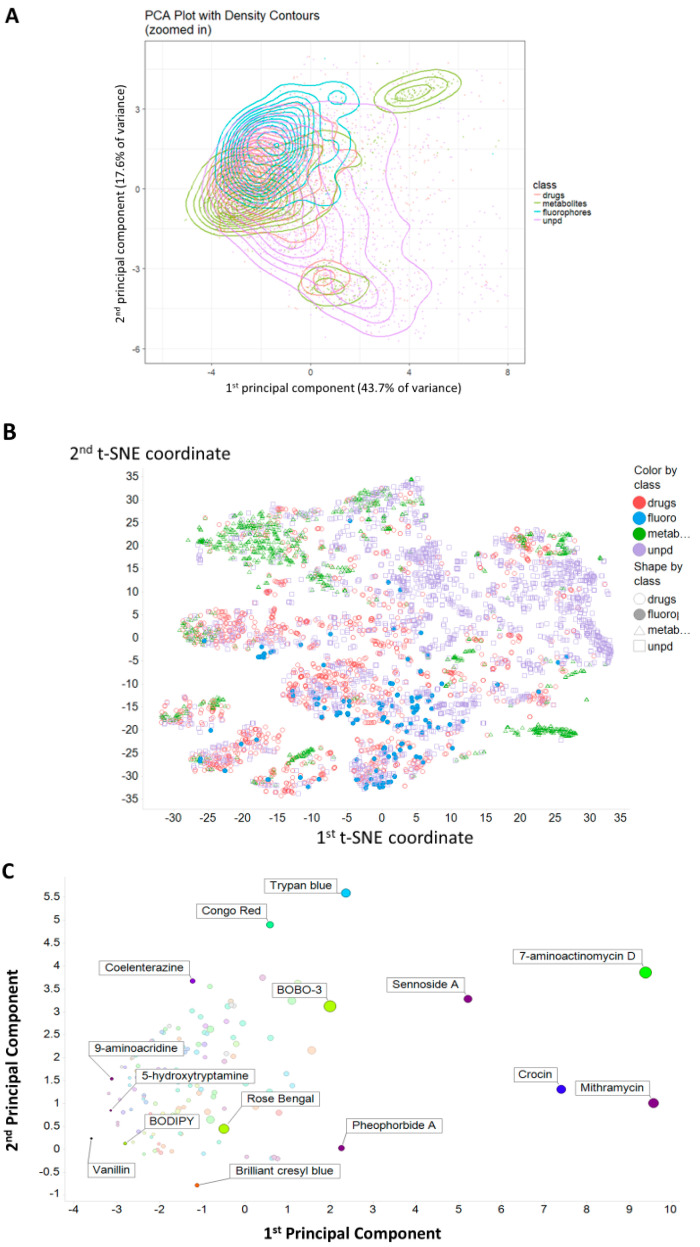
Principal components and t-SNE plots of the principal components of the variance in calculated properties of the molecules used. (**A**) The first two principal components of the variance in calculated properties of the four classes fluorophores, drugs, metabolites and natural products. Molecules are as in Supplementary Fluorophores SI.xlsx, with the drugs and metabolites those given in [45]. A sampling of 2000 natural products from our download [55] of UNPD was used. Descriptors were z-scores normalised and correlation filtered (threshold 0.98). (**B**) t-SNE plot of the data in (**A**), using the same colour-coding. (**C**). Plot of the first two principal components of the variance in the fluorophores alone. The excitation wavelength is encoded in the colour of the markers. The size of the symbol encodes the molecular weight, indicating that much of the first PC is due to this (plus any other covarying properties).

Figure 1A shows a Principal Components Analysis (PCA) plot of the distribution of the four classes based on a series of descriptors in RDKit (www.rdkit.org/), while Figure 1B shows a t-SNE [82] plot of the same data. These clearly show a strong overlap between the rather limited set of fluorophores used and quite significant parts of the drug space. Figure 1C gives just the fluorophores, with the nominal excitation maximum encoded in its colour. This suggests that even with just ~150 molecules, we have achieved a reasonable coverage of the relevant ‘fluorophore space’, with no obvious bias or trend in excitation wavelengths.

**Figure 2:**
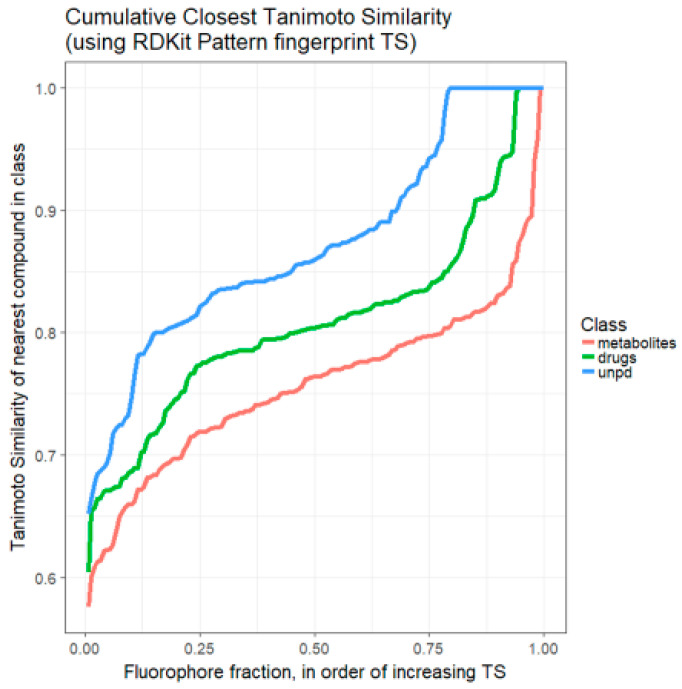
Ranked order of Tanimoto similarity for fluorophores vs. marketed drugs (green line/**—**), fluorophores vs. Recon2 metabolites (red line/**—**), and fluorophores vs. a 2000-member sampling of UNPD (blue line/**—**). Each fluorophore was encoded using the RDKit ‘Patterned’ encoding, then the Tanimoto similarity for it was calculated against each drug, metabolite or natural product sample. The highest value of TS for each fluorophore was recorded and those values ranked. Read from right to left.

We previously developed the use of rank order plots for summarising the relationships (in terms of Tanimoto similarities) between a candidate molecule or set of molecules and a set of targets in a library [45]. Figure 2 shows such a rank order plot, ranking, for each fluorophore, the most similar molecule in the set of endogenous Recon2 [45,83] metabolites, the set of marketed drugs [45], and a random subset of 2000 of some 150,000 molecules taken [55,84] from the Unified Natural Products Database (UNPD) [85]. This again shows very clearly that the majority of fluorophores chosen do look moderately similar (TS > 0.75) to at least one drug (and even more so to representatives of the natural products database).

**Figure 3:**
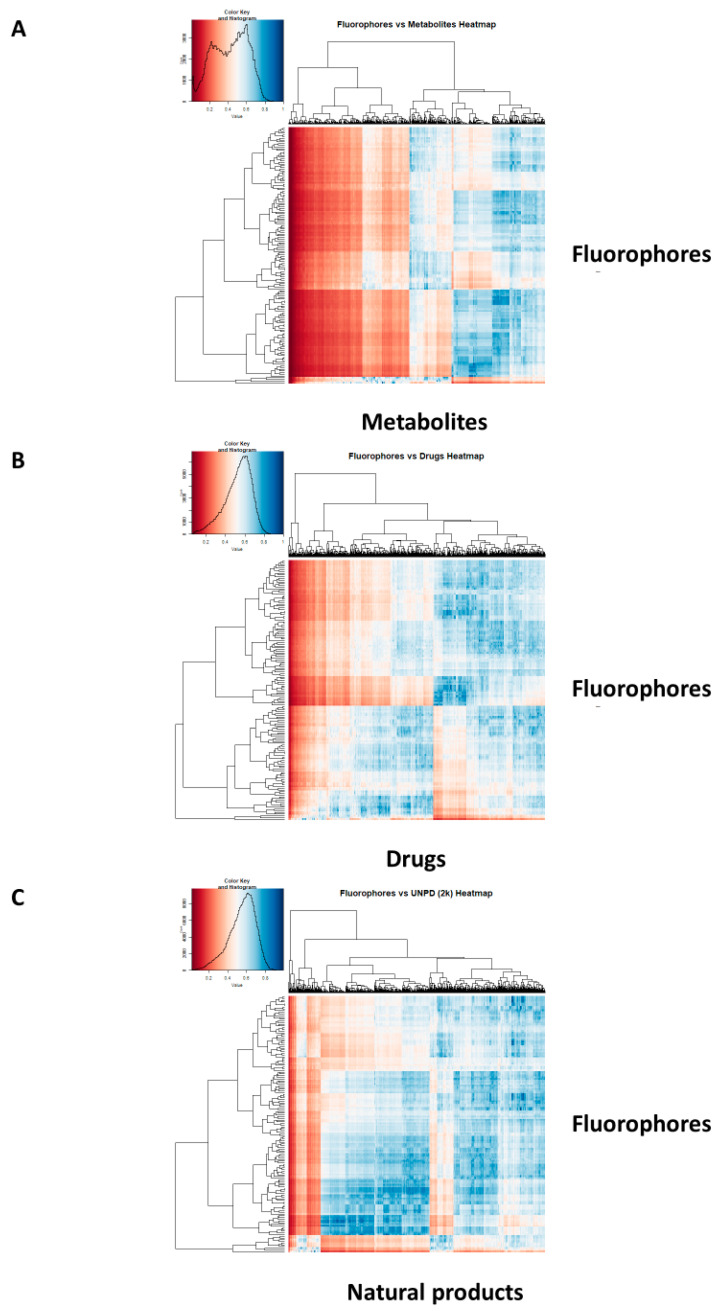
Heat maps illustrating the Tanimoto similarities (using the RDKit patterned encoding) between our selected fluorophores and (**A**) Recon2 metabolites, (**B**) Drugs, and (**C**) a subset of 2000 natural products from UNPD.

It is also convenient [45] to display such data as a heat map [86], where a bicluster is used to cluster similar structures and the colour of the cell at the intersection encodes their Tanimoto similarity. Figure 3 shows such heatmaps for fluorophores vs. (A) endogenous (Recon2 [87]) metabolites, (B) drugs, and (C) 2000 sampled natural products from UNPD. The data reflect those of Figure 2, and it is again clear that for each fluorophore there is almost always a drug or a natural product for which the average Tanimoto similarity is significantly greater than 0.7.

While it is rather arbitrary, to say the least (given how the Tanimoto similarity varies with the encoding used), as to whether a particular chemical structure is seen by humans as ‘similar’ to another, we provide some illustrations that give a feeling of the kinds of similarity that may be observed.

**Figure 4:**
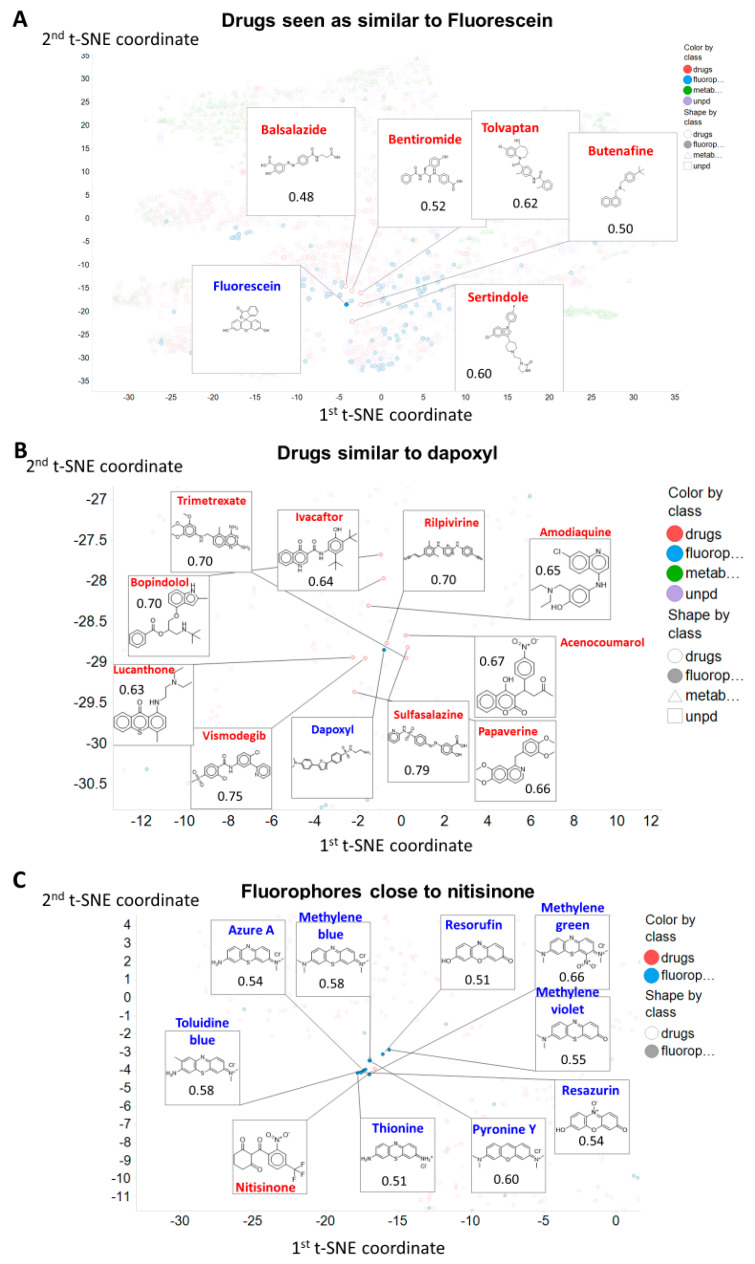
Observable structural similarities between selected fluorophores and drugs. The chosen molecules are (**A**) fluorescein, (**B**) dapoxyl (both fluorophores) and (**C**) nitisinone (a drug). Data are annotated and/or zoomed from those in Figure 1B.

Thus (Figure 4A) we illustrate the drugs closest to fluorescein in the t-SNE space (as per Figure 1B), since fluorescein is a very common fluorophore, is also widely used in ophthalmology (e.g., [88,89]), and can enter cells via a variety of transporters [90] such as monocarboxylate transporters (SLC16A1, SLC16A4) [91], SLCO1B1/3B1 [92,93] and SLC 22A20 [94] (see also Table 1).

Fluorescein is similar in t-SNE space (Figure 4A) to a variety of drugs. This similarity is not at all related to the class of drug, however, as close ones include balsalazide (an anti-inflammatory used in inflammatory bowel disease [95]), bentiromide (a peptide used for assessing pancreatic function [96]), butenafine (a topical antifungal [97]), sertindole (an atypical antipsychotic), and tolvaptan (used in autosomal dominant polycystic kidney disease [98]). Similar remarks may be made of dapoxyl (Figure 4B). Note, of course, that the t-SNE plots are based on property descriptors, while the Tanimoto distances are based on a particular form of molecular fingerprint, so, *a priori*, we do not necessarily expect the closest molecules to be the same in the two cases. In addition, we note that molecules with different scaffolds may be quite similar; in the cheminformatics literature, this is known as ‘scaffold hopping’ (e.g., [99,100,101,102,103,104]).

For a drug, we picked nitisinone, a drug active against hereditary tyrosinaemia type I [105] and alkaptonuria [106,107], as it is surrounded in t-SNE space (Figure 4C) by several tricyclic fluorophores, that do indeed share similar structures (Figure 4C).

**Figure 5:**
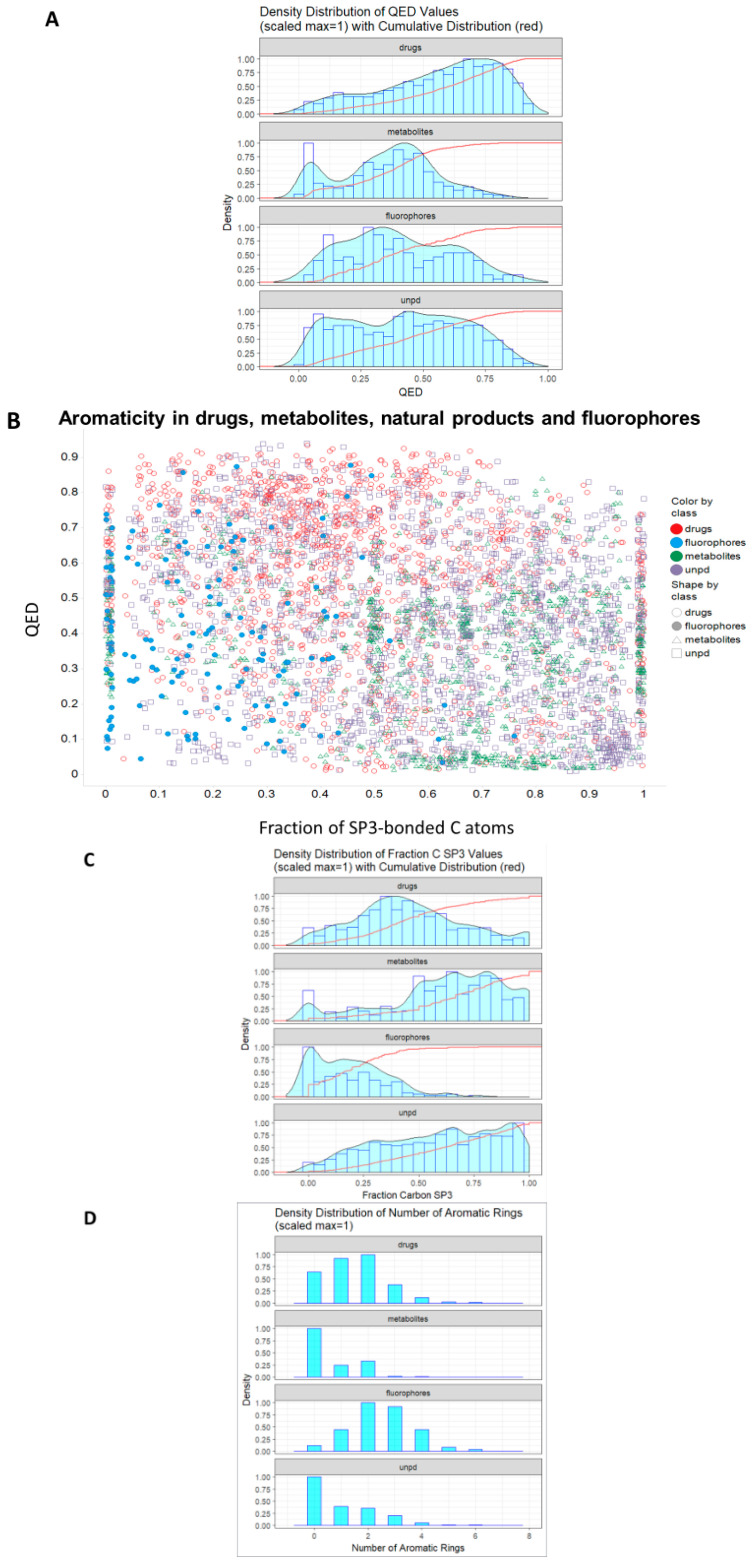
Distribution of quantitative estimate of drug-likeness (QED) values in different classes of molecule. (**A**). Cumulative distributions for the four classes. (**B**). Relationship between QED and aromaticity for the four classes as encoded by the fraction of C atoms exhibiting sp^3^ bonding. QED values were calculated using the RDKit Python code as described in Methods and plotted in (**A**) using ggplot2 and in (**B**) using Spotfire. (**C**). Density distribution of fraction of C atoms with sp^3^ bonding. (**D**). Histogram of distributions of numbers of aromatic rings in the four given classes.

Bickerton and colleagues [108] introduced the concept of the quantitative estimate of drug-likeness (QED) (however, see [109]), and it is of interest to see how ‘drug-like’ our four classes of molecule are based on their criteria. Figure 5A shows the distribution of QED drug-likenesses for marketed drugs, for Recon2 metabolites, for our selected fluorophores, and for a sample of 2000 molecules from UNPD. Our fluorophores are noticeably more similar to drugs than are endogenous metabolites, and roughly as similar to drugs as are natural products (Figure 5A).

Given that essentially all drugs are similar to at least one natural product [55], this is entirely consistent with our thesis that most fluorophores do look rather like one or more of the marketed drugs. One aspect in which (a) drugs and fluorophores differ noticeably from (b) metabolites and natural products is the extent to which they exhibit aromaticity, encoded here (Figure 5B, on the abscissa) via the fraction of carbon atoms showing sp^3^ hybridisation (i.e., non-aromatic). This is shown as a distribution in Figure 5C. There is clearly a significant tendency for drugs to include (planar) aromatic rings, and although this is changing somewhat [110,111,112,113,114], there are strong thermodynamic reasons as to why this should be so (see Discussion). The modal number of aromatic rings for both drugs and fluorophores is two, significantly greater than that (zero) for metabolites and for natural products (Figure 5D). One reason for fluorophores to exhibit aromaticity is simple, as reasonable visible-wavelength fluorescence in organic molecules relies greatly on conjugation (e.g., [115]), to which aromatic rings can contribute strongly. This argument alone probably accounts in large measure for the drug-likeness of fluorophores.

Finally, a very recent, principled, and effective clustering method [116,117], representing the state of the art, is that based on the Uniform Manifold Approximation and Projection (UMAP) algorithm. In a similar vein, and based on the same descriptors as used in the t-SNE plots, we show the clustering of our four classes of molecule in UMAP space, where most clusters containing drugs also contain fluorophores. Despite being based on property descriptors, the UMAP algorithm is clearly very effective at clustering molecules into structurally related classes.

## 3. Discussion

Most drugs can act (often deeply) within the target organism or tissue, and thus the means by which they get to their sites of action is significant. This is considered especially true for natural products which (as with many drugs) normally do not adhere to the ‘rule of 5’ [76,118,119,120,121,122,123,124,125,126,127]. The chief answer to the question of how drugs do get through biomembranes is ‘by using SLCs’, and so it would be desirable to have high-throughput methods to assesses the activities of these transporters. Among the commoner approaches are methods that assess the uptake of fluorophores, but these are likely to ‘work’ only if drugs and natural products, including marine drugs, do in fact structurally resemble fluorophores.

The basis of the main idea presented and tested here is that the structures of common fluorophores are in fact sufficiently similar to those of many drugs (including natural products) as to provide suitable surrogates for assessing their uptake via solute carriers of the SLC (and, indeed, their efflux via ABC) families. While the latter transporters are well known to be rather promiscuous, and to transport a variety of fluorophores [40,42,128,129,130], considerably less attention has been paid to the former. As mentioned in the Introduction, some marketed pharmaceutical drugs that are transported into cells are in fact naturally fluorescent, including anthracyclines [56,57,58], mepacrine (atebrin, quinacrine) [59], obatoclax [60,61], tetracycline derivatives [57,62] and topotecan [63], while the same is true of certain vitamins riboflavin [64,65] and certain bioactive natural products (e.g., [66,67,68]). As an illustration, and as a complement to our detailed gene knockout studies [23], Table 1 gives an indication of dyes whose interaction with specific transporters has been demonstrated directly. In some cases, their surrogacy as a substrate for a transporter with a known non-fluorescent substrate is clear, and as mentioned in the introduction, they are sometimes referred to as ‘false fluorescent substrates’. Overall, while not intended to be remotely exhaustive, this Table does serve to indicate the potentially widespread activity of transporters as mediators of fluorophore uptake, and indeed, a number of such transporters are known to be rather promiscuous.

**Table 1:** Some examples in which fluorescent dyes have been found to interact with uptake transporters directly as substrates or inhibitors. We do not include known non-fluorescent substrates to which a fluorescent tag has been added (see, e.g., [131,132,133]).

Dye	Transporter	Comments	Reference
Amiloride	OCT2 (SLC22A2)	A drug. Rhodamine 123 and 6G also served as substrates.	[134]
4′,6-diamidino-2-phenylindol (DAPI)	OCT1 (SLC22A1)	Potently inhibited by desipramine and also by various organophosphate pesticides.	[135,136]
DiBAC(4)3	Na^+^/HCO3^−^ NBCe1-A SLC4A4	Competes with 4,4′-Diisothiocyanatostilbene-2,2′-disulfonic acid	[137]
5-carboxyfluorescein	OAT3 (SLC22A8	Very high V_max_	[94]
6-carboxyfluorescein	OAT1 (SLC22A6)		[94,138]
2′,7′-dichlorofluorescein	OATP1B1 (SLCO1B1)	Good substrate	[139]
4-(4-(Dimethylamino) styryl)-N-methylpyridinium (ASP^+^)	Dopamine transporter (SLC6A3)		[140,141]
	Noradrenaline transporter (SLC6A2)		[140,142,143]
	Serotonin transporter SLC6A4		[140,144]
	Various monoamine transporters		[145]
	OCT1/OCT2 (SLC22A1/2);	Seen as a model substrate	[146]
	Various OCT transporters		[147]
	Other, unknown (non-OCT1/2) transporters with low affinity		[148]
Ethidium	OCT1/2/3 (SLC22A1/2/3)	Substrate	[149]
FFN511	VMAT2 (SLC18A2)	‘False fluorescent neurotransmitter’ (i.e., surrogate substrates) concept	[79]
FFN54/246	SLC6A4, SLC18	‘False’ fluorescent substrates for serotonin and VMAT transporters. Potent inhibition by imipramine and citalopram	[80]
FFN270	SLC6A4, SLC18	Another example of a fluorescent false neurotransmitter	[150]
Fluorescein	SLCO1B1/3B1	Effective substrate; analysis of inhibitors	[92,93]
	OAT6 (SLC22A20)		[94]
	SLC16A1, SLC16A4		[91]
	Many OATPs (SLCO family) expressed in insect cells		[90]
Lucifer yellow	Sodium-dependent anion transporters	Inhibited by probenecid	[151,152]
Rhodamine 123	OCT1/OCT2 (SLC22A1/2)	Potent substrate	[153]
Stilbazolium dyes	Norepinephrine transporter (SLC6)	Dyes related to ASP^+^	[154]
Zombie Violet, Live/Dead Green, Cascade Blue, Alexa Fluor 405	OATP (SLCO) 1B1/1B3 and 2B1	All shown to be direct substrates, and uptake inhibited by known transporter inhibitors	[155]

**Figure 6:**
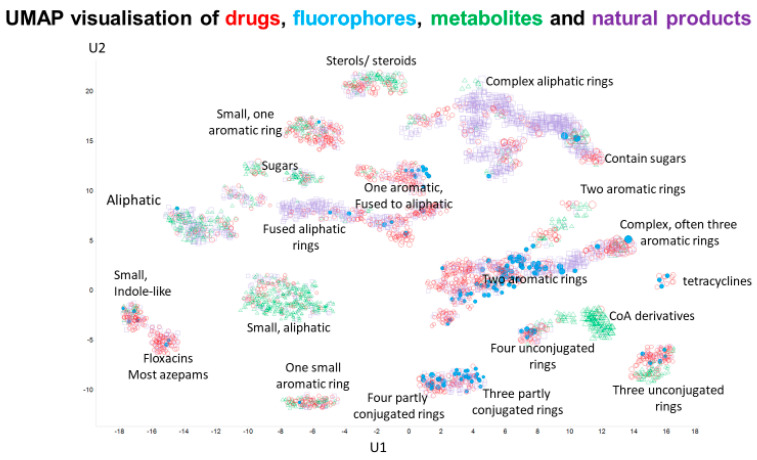
UMAP projection into two dimensions of the four classes of molecules, annotated by the type of molecular structure in the various clusters.

Structural similarity (or the assessment of properties based simply on analyzing structures) is an elusive concept (e.g., [156,157]), but as judged by a standard encoding (RDKit Patterned), there is considerable similarity in structure between almost all of our chosen fluorophores and at least one drug, whether this is judged by their descriptor- or fingerprint-based properties (Figure 1, Figure 2 and Figure 3), by observation (Figure 4 and Figure 6), or (Figure 5) via the QED [108] measure.

Although there is a move towards phenotypic screening [158,159,160,161], many drugs were developed on the basis of their ability to bind potently in vitro to a target of interest. If the unbound molecule is conformationally very flexible, and the bound version is not, binding necessarily involves a significant loss of entropy. Potent binding (involving a significant loss in free energy) of such a molecule would thus require a very large enthalpic term. Consequently, it is much easier to find potent binders if the binding can involve flat (which implies aromatic), conformationally inflexible planar structures. Such reasoning presumably reflects the observation (Figure 5B) that drugs tend to have a low sp^3^ character, typically with a number of aromatic rings. Conjugated aromatic rings are also a major (physical and electronic) structure that allow fluorescence from organic molecules [162,163,164,165], with greater π-bond conjugation moving both absorbance and fluorescence toward the red end of the spectrum. Overall, these two separate roles for aromatic residues, in low entropy of binding and in electronic structure, provide a plausible explanation for much of the drug-likeness of common fluorophores.

While this study used a comparatively small set of fluorophores, increasing their number can only increase the likelihood of finding a drug (or natural product) to which they are seen to be similar. This said, this set of molecules provides an excellent starting point for the development of competitive high-throughput assays of drug transporter activity.

## 4. Materials and Methods

Fluorophores were selected from the literature and by scanning various catalogues of fluorophores, and included well known cytochemical stains, food dyes, laser dyes and other fluorophores, including just a few marketed drugs plus fluorescent natural products. We chose only those whose structures were known publicly. The final set included 150 molecules. Supplementary Fluorophores SI.xlsx gives a spreadsheet of all the relevant data that we discuss, including the marketed drugs, Recon2 metabolites [87] (both given also in Reference 37) and a subset of 2000 natural products from UNPD (see [55,85]).

Although there are a great many possible molecular encodings (whether using molecular fingerprints or vectors of calculated properties), each of which can give a different Tanimoto similarity, for our present purpose we chose to use only the Patterned encoding within RDKit (www.rdkit.org/). We also used the RDKit version of QED (https://www.rdkit.org/docs/source/rdkit.Chem.QED.html). Workflows were written in KNIME as per our standard methods [45,46,47,48,55,84,166,167]. t-SNE plots used the first 10 PCs (95.3% explained variance) as inputs based on 27 RDKit descriptors, and were otherwise as previously described [168].

## 5. Conclusions

An analysis of some 150 fluorophores in common usage in biological research has shown that a great many of them bear significant structural similarities to marketed drugs (and to natural products). This similarity holds true whether the analysis is done using structures encoded as fingerprints or via physico-chemical descriptors, by visual inspection, or via the quantitative estimate of drug likeness measure. For any given drug, there is thus likely to be a fluorophore or set of fluorophores that is best suited to competing with it for uptake, and thus for determining, by fluorimetric methods, the QSAR for the relevant transporters. This should provide the means for rapid and convenient competitive and trans-stimulation assays for screening the ability of drugs to enter cells via SLCs.

## Supplementary Materials

The following are available online at https://www.mdpi.com/1660-3397/18/11/582/s1. Fluorophores SI.xlsx: The list of all the molecules and properties illustrated in the present analysis. The descriptors are taken from rdkit, and each of them is explained in detail, with relevant literature references, at https://www.rdkit.org/docs/GettingStartedInPython.html#list-of-available-descriptors.

## Abbreviations

PCA	Principal Components Analysis
QED	Quantitative Estimate of Drug-likeness
QSAR	Quantitative Structure–Activity Relationship
SLC	solute carrier
TS	Tanimoto similarity
UNPD	Universal Natural Products Database
